# Cannula detachment—a solution to a significant patient safety and health economic issue

**DOI:** 10.1038/s41433-026-04428-x

**Published:** 2026-03-24

**Authors:** Amar Alwitry

**Affiliations:** Ramsay Woodthorpe Hospital, Nottingham, UK

**Keywords:** Health care, Risk factors

Cannula detachment during cataract surgery is a well-recognised phenomenon resulting in entirely avoidable harm and visual loss.

This issue has been reported extensively in the literature and has been the subject of two recent safety alerts by the Royal College of Ophthalmologists in England [https://www.rcophth.ac.uk/news-views/ophthalmic-safety-alert-detachment-of-cannulas-during-ophthalmic-surgery/]. The safety alert states: “The NHS Improvement national patient safety team have informed the College of the *continued trend* of incidents involving issues with detachment of cannulas during ophthalmic surgery (cannula-associated ocular injury, COI).”

The harm can range from a simple angle bleed or iris damage, through posterior capsule rupture, to retinal damage and retinal detachment. The consequences can be blinding, even potentially resulting in loss of the eye from ensuing complications, and it is completely avoidable and indefensible.

A recent survey facilitated by the United Kingdom and Ireland Society of Cataract and Refractive Surgeons (UKISCRS) found that this happens quite frequently [1]. 84% of responding surgeons had experienced detachment of the cannula during surgery; 78% had seen harm; and 50% indicated that the last time it occurred there was ocular damage. 23% indicated that it happened on average once per year; 38% twice per year; 16% three times per year and 7% four or more times per year. **87%** **felt that a safety device was required.**

Assuming 2,000 surgeons in the UK, then 22.95% experiencing it once per year equates to 459 detachments per year. If we further extrapolate the data then there are at least 4,256 detachments annually in the UK alone. In half, harm occurs, meaning 2,128 episodes of harm occurring each year due to this avoidable phenomenon.

Assuming the commonest significant complication is posterior capsule rupture (PCR) and that this occurs in a conservative 15% of incidences, then 320 cases of PCR a year are occurring due to this complication.

Not only is the visual loss of concern, there are additional costs to the provider of caring for these patients. One study [2] estimated an extra cost of £815 per patient, meaning £261 K extra annual healthcare costs in the UK alone. If we assume a very conservative only 1in 50 lose sight because of the complication and litigate, then circa 21 cases per year litigate and seek legal redress for clinical negligence. With an estimated average compensation pay out of £100 K to the patient and legal fees of circa £200 K (combined defendant and claimant solicitor fees) then the cost per case to the UK health economy is circa £300 K per year per patient, meaning £6.3 M pay outs per year. Adding the expenditure due to the extra care/clinical costs required means an extra total healthcare bill to the UK of £6.562 M per year. If we extrapolate these findings to the worldwide health economy, then it becomes a significant issue.

For 19 years since substantive publications on this issue emerged there has been no solution available and, from a medico-legal perspective, it is indefensible.

We are currently using syringes and cannulas with a known risk, and we need a solution which is cheap, easy to apply to all our syringes, fast to use, and effective. It is important that viscoelastic manufacturers and phaco pack providers engage in safety work to try and remedy the situation by utilising/developing a safety device and take some responsibility for providing devices with a known safety flaw.

Working with manufacturers we have developed a paper-based solution to the issue which should become compulsory for all intraocular procedures worldwide. It consists of a piece of sterile medical tape with a hole in it. The cannula is attached to the Luer lock syringe as per usual practice and then the cannula is passed through the hole in the tape. The ends of the tape are then secured to the sides of the syringe thus rendering it safe. If the cannula does detach it cannot shoot into the eye and thus the harm and visual loss is avoided.

**Fig. 1:**
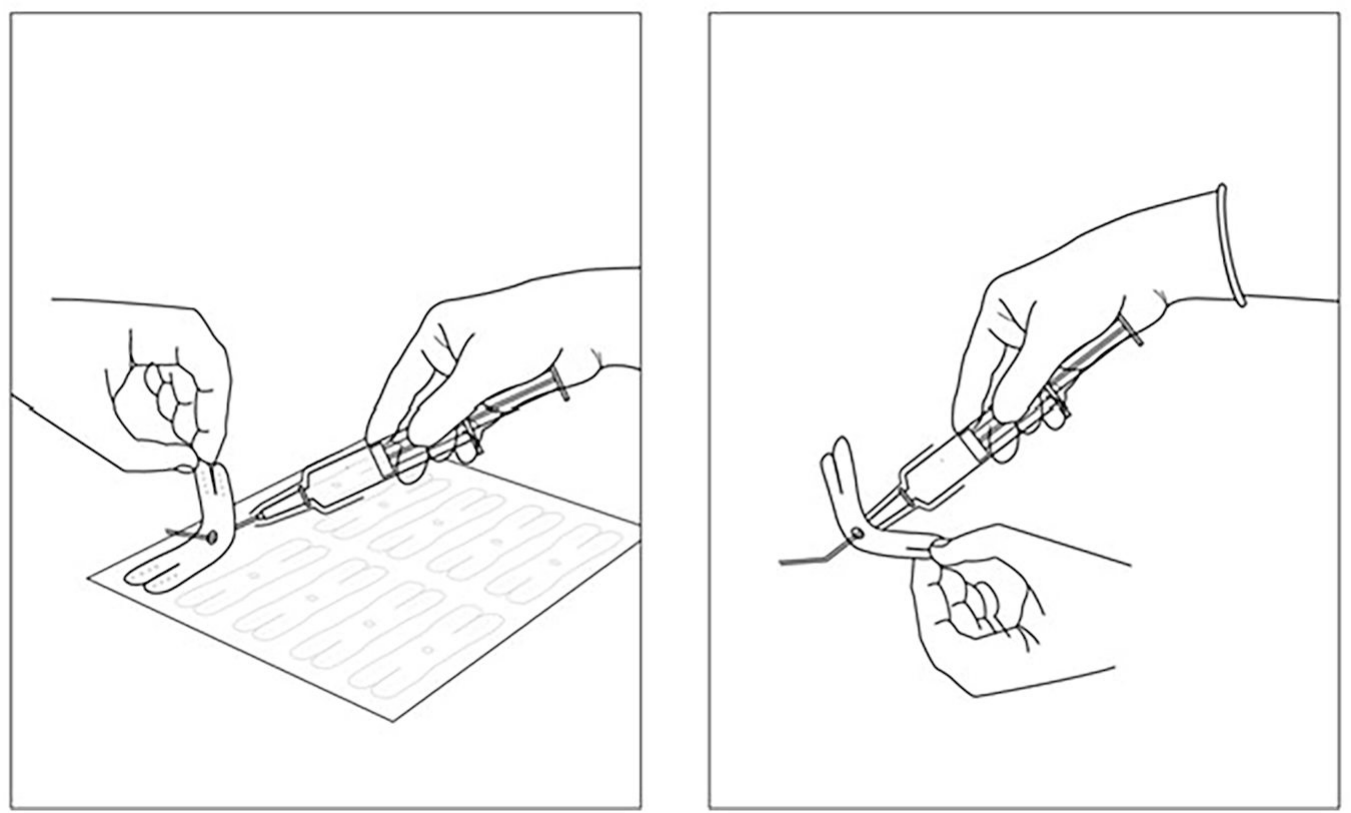
Application process is shown whereby the tape is lifted from the carrier sheet and the cannula passed through the hole. The clear adhesive legs of the tape are attached to the syringe sides rendering it safe.

Figure 1 shows the application of the tape. The tape is lifted from the sheet and the hole exposed. The cannula is then passed through the hole ensuring that fingers are kept clear. The clear adhesive legs of the tape are simply attached to the body of the syringe. It can be applied to any syringe size and utilised with any cannula or needle.

It is applied by the scrub practitioner as part of their preparations before the case commences and takes a moment to apply to all the syringes therefore, importantly, it does not increase surgical time or involve the operating surgeon at all. It is easily removed and does not increase plastic wastage. It should be applied at the start of the procedure and form part of the surgical safety checklist. In line with questioning about the sterility of the equipment there should be an additional question “are the cannulae secure?”.

The use of this tape will abolish this complication, have significant health economy benefits, lower litigation costs, remove the finger of blame on the surgeon and scrub practitioner for poor surgical practice, and, most importantly, prevent avoidable visual loss in patients who would have otherwise achieved a perfect surgical outcome.

With such a simple, cheap and effective safety solution available, it should be used for all intraocular procedures and failure to do so would, in my view, and, I believe in the view of the Courts worldwide, be deemed to be a breach of the duty of care to the patient. The safety of our patients has to be paramount and a solution such as this one presented or something similar should be adopted as a matter of routine.
